# Supplementary material for: Are carbon emissions trading and green financial instruments synergistic? -Comprehensive quantitative research based on content analysis
Source: PLoS One. 2024 Mar 7;19(3):e0298601. doi: 10.1371/journal.pone.0298601 (PMC10919601; doi:10.1371/journal.pone.0298601)
Supplement: S1 Data — (ZIP) [file pone.0298601.s001.zip › Data/地级市Z_F工作报告.zip!/╣π╬≈/▒▒║ú╩╨/└┤╫╘╣1⁄2╓┌║┼í╛┬φ┐╦╩2╛▌═°í┐_2014.docx]

各位代表：

现在，我代表北海市人民政府向大会作政府工作报告，请予审议，并请各位政协委员和其他列席同志提出意见。

一、2013年工作回顾

2013年，面对宏观经济的复杂形势和下行压力，在自治区党委、政府和市委的正确领导下，全市人民紧紧围绕在广西率先全面建成小康社会的奋斗目标，坚持“真抓实干、把事干成、造福百姓”的理念，落实稳中求进的工作总基调，着力稳增长、调结构、促改革、惠民生，沉着应对风险挑战，全面推进科学发展，巩固和扩大了实施北海三年跨越发展工程的成果，全市经济在连续几年高速增长的基础上保持了优质较快发展，继续呈现出“速度快、质量好、结构优、后劲足”的鲜明特点，各项事业取得了新的进步。

经济发展取得明显成效。全市完成地区生产总值735亿元、增长13.3%，增速排全区第一位；财政收入113.6亿元、增长13.5%，增速排全区第三位，财政收入占地区生产总值的比重比全区平均水平高1.6个百分点，税收收入占财政收入的比重比全区平均水平高11个百分点，收入质量进一步提高；规模以上工业总产值1300.9亿元、增长27%，增速排全区第二位；规模以上工业增加值增长24.6%，增速排全区第一位；全社会固定资产投资685.9亿元，增长23.5%。三次产业结构调整为19.4:50.8:29.8。

优势产业发展取得明显成效。电子信息、石油化工、临港新材料三大产业合计完成产值突破千亿元大关、达到1120亿元，增长25%。我们为之努力长达12年、总投资192亿元的北海林纸一体化项目实质性开工建设；总投资177.8亿元的广西（北海）LNG项目引堤和陆域形成工程交付验收，输气管道建设抓紧推进；总投资约400亿元的神华国华广投北海能源基地项目获得国家发改委“路条”，建设工作扎实开展。三诺智慧产业园建成开园，朗科国际存储产业园一期正式投产，北海出口加工区B区基本完成封关必备项目建设任务。

交通基础设施建设取得明显成效。南宁至北海高速铁路正式运营，标志着北海迈进了高铁时代。总长132公里、投资超过50亿元的玉林至铁山港铁路全线铺通。总长14.5公里、北海有史以来建设标准最高的银滩大道改造工程完工通车。北海千里乡村公路畅通工程即将全面完成，3年来累计投入5.3亿元，建成通达500多个行政村、自然村的水泥公路1018公里，受惠群众超过60万人。

保障和改善民生取得明显成效。筹措13.6亿元的资金，全面完成了年初承诺的10件为民办实事项目。设立18年的北铁公路收费站终止收费并完成拆除，方便了群众出行。投资3.8亿元、备受市民关注的北海中学新校区投入使用。桂林电子科技大学海洋信息工程学院正式招生，结束了北海没有二本院校的历史。投资2.3亿元的平安城市二期工程即将完工，建成后北海的视频监控覆盖率将为全区地级市最高。城镇居民人均可支配收入达到23407元、增长10.4%，农民人均纯收入达到8239元、增长14%。

一年来，我们主要做了以下工作：

(一)着力发展实体经济，不断增强产业支撑。

“三农”工作扎实推进。特色现代农业蓬勃发展，完成农林牧渔业总产值212.83亿元、增长5.6%。发放农作物良种补贴、成品油价格补贴等政策性支农惠农资金15.91亿元。农业产业化步伐加快，设施农业示范基地发展到33个，农业龙头企业达到43家，农民专业合作社达到281家。粮食、蔬菜、畜禽、水产品等主要农产品产量稳定增长，林下经济和庭院经济快速发展。新农村建设扎实推进，水利固定资产投资完成8.1亿元，解决了13.61万人的饮水安全问题。扶贫攻坚力度加大，54条贫困农村屯级道路竣工，惠及群众2.87万人。加强防灾减灾能力建设，成功抗击强台风“海燕”等自然灾害。

推进新型工业化取得新进展。采取“四定”办法强力推进290个市级重点项目，完成投资137.7亿元。惠科电子科技产业园等一批重点项目开工建设，诚德镍业固溶1号及2号生产线建成投产、3号线加快建设，国投北海电厂二期等项目前期工作抓紧推进。铁山港（临海）工业区完成产值535亿元，增长29.2%；北海工业园区完成产值328亿元，增长30.2%；北海出口加工区完成产值180亿元，9月份单月进出口额首次超过1亿美元；北海高新区完成产值50亿元，软件和信息服务业、创意产业初步形成集聚效应；北海国家（海洋）农业科技园区暨海洋产业科技园区规划编制完成，中国水产科学研究院等11家知名科研机构签约入园；龙港新城（香港产业园）筹建工作扎实推进。

以旅游业为龙头的现代服务业繁荣发展。出台政策措施，努力推动旅游产业跨越发展，涠洲岛保护开发步伐加快，中越海上旅游航线复航，第五届国际珍珠节暨2013年海滩旅游文化节成功举办，旅游环境综合整治工作全面推进，接待国内游客1500万人次、增长15%，完成国内旅游收入130亿元、增长20%。北海至涠洲岛海上客运量超过160万人次，增长21.9%。机场旅客吞吐量达到84.8万人次，增长18.9%。培育发展北海至香港集装箱定期班轮航线，北海至高雄定期班轮成功开通，完成港口吞吐量2078万吨，增长18.2%。金融环境进一步优化，不良贷款余额和比重连续5年实现“双降”，小微企业贷款增长26.2%。商贸、信息、会展等服务业加快发展，第三产业增加值增长8.1%。

（二）着力加强城市建设管理，深入推进生态文明建设。

“城市建设管理年”活动取得新成效。城市总体规划修编抓紧推进，城市特色研究规划成果通过专家评审，银滩中区等区域控制性详细规划、城市道路综合交通与市政等一批专项规划编制工作启动。浙江路、贵阳路等一批城市道路，广东路和上海路铁路立交工程，北海大道风貌整治、城中村改造和一批示范街、示范路、示范小区建设稳步推进。制约上海路建设近20年、市民高度关注的徐屋东段搬迁遗留问题得到彻底解决，最后1户搬迁户完成搬迁，相关建筑物已经全部拆除。开展168条小街小巷改造，已竣工77条。高度重视市区内涝整治工作，完成茶亭路等易涝路段整治38处，投资8.1亿元的市区内涝整治二期工程加快推进。市区道路市场化保洁面积扩大至393万平方米，增长28.9%。环卫作业机扫率达到43%，比2011年提高了28个百分点。开展“四车”综合整治，实施电动自行车登记管理，规范出租车市场秩序，努力治理城市交通拥堵问题，构建联勤联管联治工作机制，加强城市管理综合整治，城市管理能力和水平进一步提高。

生态文明建设深入开展。筹措资金6.09亿元开展“美丽广西·清洁乡村”活动，出动人员66万人次，清理水源2866处、农田65.9万亩、海岸线560公里，4320个自然村配备了保洁员，乡村环境卫生状况明显好转，长效机制逐步建立。银滩中路、侨港区域和广东南路污水截流整治工程竣工，银滩沿线污水截流系统逐步完善。克服时间紧、征地难等困难，认真做好第四届广西园林园艺博览会承办准备工作，园博园建设全面铺开。加强生态环境监测保护，启动建设涠洲岛珊瑚礁国家海洋公园，实行PM2.5实时发布，深入开展环境安全隐患大清查大整治，严厉打击电捕鱼等非法行为。节能减排工作扎实推进，万元生产总值能耗下降2.8%。荣获“国家节水型城市”、“全国节水型社会建设示范区”和十大“最中国生态名城”称号，山口红树林保护区被评为“中国十大魅力湿地”，涠洲岛被评为“美丽中国”十佳海洋旅游目的地。

(三)着力推进改革创新，不断深化开放合作。

整合工商、质监、商务等部门职能，推进食品药品安全监管体制改革，取得了突破性进展；合浦县人民医院等4家县级公立医院列入全区第二批改革试点，医药卫生体制、“营改增”等改革扎实推进；市外贸总公司等6家国有企业完成改制。对内对外开放通道逐步完善，玉林至铁山港高速公路投入使用，贵港至合浦高速公路项目加快推进，合浦至湛江铁路项目建议书获国家发改委批复。铁山港口岸获国家批准实行临时开放，正式扩大开放完成审理阶段工作，5、6号泊位建设获得核准。积极开展与著名高校的战略合作，与清华大学签订全面合作协议。主动参与北部湾经济区同城化、南北钦防玉崇百七市一体化，加强旅游、物流、交通等方面的合作。招商引资取得新成效，新引进和润粮油加工等一批重大项目，与香港中华厂商联合会签订战略合作框架协议。组团参加“2013美丽广西港澳行”活动，签约项目总投资额达24.32亿美元。推进电子商务平台建设，获批为广西电子商务示范基地和服务外包园区。引进项目实际到位资金374亿元，实际利用外资3.2亿美元。完成外贸进出口总额27亿美元，增长29.9%。

(四)着力发展社会事业，全面加强社会建设。

科技自主创新能力稳步提高，首个院士工作站投入运行，北部湾电子信息孵化器累计入驻中小企业达113家。海城区十八小、银海区一小建成投入使用，北师大附中、北海十中、银海区二小等一批中小学校加快建设，主城区初中一年级“大班额”问题得到有效解决。高等教育事业加快发展，北海艺术设计职业学院升本设置通过教育部专家组初审。文化惠民工程深入实施，村级公共服务中心新增9个，总数达到135个；全国重点文物保护单位新增3处，总数达到7处；“海上丝绸之路”申遗工作扎实推进，市非物质文化遗产展示中心建成并向公众免费开放；大型舞剧《碧海丝路》获得第十四届文华奖“优秀剧目奖”，在国家大剧院成功进行商演。"120"急救网络体系、全科医生临床培训基地、合浦中医院等一批卫生项目加快建设，合浦县人民医院门诊大楼建成投入使用。竞技体育取得良好成绩，群众体育蓬勃发展，合浦县荣获“全国群众体育先进单位”称号。人口计生目标管理任务全面完成。社会保险待遇和最低生活保障水平稳步提高，企业退休人员基本养老金人均每月增加186元，城镇和农村低保人均补助水平高于自治区标准。新农合参合率达到99.32%，继续名列全区首位。新开工建设保障性住房项目17个、3730套，分配入住保障性住房3067套，新增发放廉租住房租赁补贴631户，完成农村危房改造3701户，一批困难群众的居住条件得到明显改善。城镇新增就业3.15万人，登记失业率3.15%、低于目标控制数。新增农村转移就业人口1.61万人。完成社会消费品零售总额167亿元，增长14%。推进社会管理创新，积极消除影响社会稳定因素，加快处置信访积案，切实预防和有效化解群体性事件，严厉打击“两抢一盗”和传销等违法犯罪行为，各类刑事案件发生数同比下降18%。安全生产形势持续稳定好转，食品药品监管力度加大。基层应急能力稳步提高，突发事件得到有效处置。

(五)着力加强政府自身建设，不断提升政务服务水平。

认真践行党的群众路线，严格落实“八项规定”和“约法三章”精神，坚决整治“四风”，扎实开展“三服务”、“四清理”。坚持建章立制，完善《政府工作规则》和配套制度，规范政府合同管理，加大监察审计、政务督查监督力度，全面推进各项工作机制化、制度化和规范化。坚持发扬“两脚泥”精神，主动作为，勤奋工作，切实提高政府执行力。厉行勤俭节约，全市“三公”经费支出下降20%。大力精简文件会议，制发文件数量比上年减少21%，召开的全市性会议次数减少58%。全面落实党风廉政建设责任制，政府系统的惩治和预防腐败体系建设深入推进。深化政务公开和政府信息公开，政府门户网站信息发布量等主要指标稳居全区首位。认真执行市人大及其常委会的各项决议，坚持向人大报告工作制度和向政协通报工作制度，主动接受市人大的法律监督、工作监督和市政协的民主监督。广泛听取市人大、市政协，以及各民主党派、工商联、人民团体和社会各界人士意见建议，办理人大代表建议45件、政协提案332件，办结率和满意率均达100%。坚持依法行政，认真执行政府常务会议学法制度，完善行政执法监督机制，90%以上的执法部门细化了行政处罚裁量标准。推进简政放权，清理下放行政审批项目186项。在全区率先开展建设工程审批流程优化改革，项目审批时限缩短60%以上。全面启动公共资源交易监管体制改革，推行建筑市场、土地交易、政府采购等公共资源交易活动统一集中管理。市政务服务中心窗口受理事项17.37万件，办结17.36万件、增长19.2%，平均办件承诺时间比法定时间缩短了3.65个工作日，群众满意率达99.97%。推动政务服务政务公开向基层延伸，195个行政村(社区)建立了政务服务中心。

一年来，外事、侨务、统计、海事、档案、地方志等工作取得新进展，妇女儿童等事业取得新进步。国防动员和后备力量建设、海防管控和基础设施建设进一步加强，双拥共建工作取得新成绩。

各位代表！我们深知，过去一年的成绩来之不易。这是自治区党委、政府和市委正确领导、科学决策的结果，是市人大及其常委会和市政协有效监督、大力支持的结果，是历届班子历任领导打下良好基础的结果，是全市人民共同奋斗的结果。我谨代表北海市人民政府，向在座的人大代表、政协委员，向全市干部群众、离退休老同志，向驻市人民解放军、武警部队官兵，向各民主党派、工商联、人民团体和无党派人士，向所有关心支持北海发展的港澳台同胞和海内外朋友，表示衷心的感谢和崇高的敬意！

我们清醒地认识到，经济社会发展和政府工作还存在不少困难和问题，主要是：现代服务业发展相对滞后，消费增长乏力，产业发展中不平衡、不协调的问题还比较突出；项目前期工作不够扎实，少数重点项目推进不快；市区部分路段内涝和交通拥堵问题依然突出，城市综合管理水平有待进一步提高；优质教育医疗资源依然不足，基本公共服务发展不够均衡，民生领域还有不少问题急需解决；行政审批效率和服务有待进一步提高，政府部门少数公务员作风不实、创新能力不足、执行力不强，与科学发展的要求还不相适应。我们一定要以高度负责的态度，不断克服困难、解决问题，努力把各项工作做得更好。

二、2014年的主要目标和任务

今年是贯彻落实党的十八届三中全会精神、全面深化改革的开局之年，也是实现“十二五”发展目标至关重要的一年。做好今年的工作，有着十分重大而深远的意义。今年政府工作的总体要求是：以邓小平理论、“三个代表”重要思想、科学发展观为指导，深入贯彻党的十八大、十八届三中全会及中央经济工作会议精神，全面落实自治区党委、政府及市委的决策部署，坚持“真抓实干、把事干成、造福百姓”的理念，坚持走“产业带动跨越发展、文化引领持续繁荣、开放增添不竭动力”的发展路子，以改革开放促创新发展，加快经济结构调整优化，围绕加强和巩固农业基础地位加快发展特色现代农业，以打造千亿元产业为重点加快新型工业化跨越发展，以加快旅游产业跨越发展为重点繁荣发展现代服务业，以推进新型城镇化为重点加快城乡一体化发展步伐，切实保障和改善民生，推进生态文明建设，奋力开创北海改革开放和科学发展新局面，加快在广西率先全面建成小康社会步伐。

今年经济社会发展的主要预期目标是：地区生产总值增长12%、达到820亿元；财政收入增长12%、达到127亿元；固定资产投资增长20%、达到780亿元；规模以上工业总产值增长26%、达到1650亿元，增加值增长21%；城镇居民人均可支配收入增长11%、达到25900元；农民人均纯收入增长13%、达到9300元；居民消费价格涨幅指数控制在4%左右；全面完成自治区下达的节能减排控制性任务。

为实现上述目标，我们将重点做好以下工作：

(一)加快深化改革扩大开放，不断增强经济社会发展的动力。

努力推进全面深化改革，积极先行先试，增强改革的系统性、整体性、协同性，力争在重点领域和关键环节改革上取得新突破。坚定不移地实施更加积极主动的开放带动战略，广泛凝聚发展要素，努力增强发展动力。

深化农村改革。推动农村产权制度改革，推进农村承包地、宅基地、林地、住房确权登记颁证工作，积极稳妥有序推进土地集中流转，推进农村产权流转交易市场建设。按国家政策赋予农民对承包地占有、使用、收益、流转及承包经营权抵押、担保权能，允许农民以承包经营权入股发展产业化经营。加快构建新型农业经营体系，坚持家庭经营在农业中的基础性地位，推进农业经营方式创新。稳定农村土地承包关系并保持长久不变，在坚持和完善最严格的耕地保护制度前提下，鼓励发展多种形式规模经营。鼓励和引导工商资本到农村发展适合企业化经营的现代种养业，向农业输入现代生产要素和经营模式。鼓励社会资本投向农村建设，允许企业和社会组织在农村兴办各类事业。继续推进林业、水利、气象等各项改革。

深化城镇化发展体制机制改革。积极拓宽城镇建设融资渠道，允许社会资本通过特许经营等方式参与城镇基础设施投资和运营。深化城镇管理改革，整合资源，理顺体制，形成合力，逐步完善交通、市政、路网、管线、市容市貌、卫生保洁、户外广告和噪音、臭气、臭水污染等方面的管理治理长效机制。统筹城乡基础设施建设和社区建设，推进城乡基本公共服务均等化，加快建立健全以工促农、以城带乡、工农互惠、城乡一体的体制机制。深化城镇户籍制度改革，推进全面放开建制镇落户限制。建立和完善参与区域城市发展协调机制，主动融入北部湾城市群建设，加快推进北部湾经济区同城化，重点抓好通信、交通、产业、城镇体系、旅游服务、金融服务、教育资源、人力资源社会保障、口岸通关等九大领域的同城化建设。

大力发展非公有制经济。大力鼓励、支持、引导非公有制经济发展，坚持权利平等、机会平等、规则平等，废除对非公有制经济各种形式的不合理规定，进一步清除市场准入障碍，消除各种隐性壁垒，拓宽投融资渠道，优化发展软环境，落实财税、金融、土地、政府采购等扶持政策，特别是积极协调解决中小微企业融资难问题，激发非公有制经济活力和创造力，推动非公有制经济跨越发展。

深化财税金融改革。积极开展“营改增”和消费税改革试点，改进预算管理，建立事权和支出责任相适应的制度，强化预算绩效管理，让公共财政更好地为人民谋福利。积极参与沿边金融综合改革试验区建设，加快金融产品和服务方式创新，完善多层次金融市场体系，努力破解中小企业融资难、融资贵的问题，加快金融事业发展。深化投融资体制改革，研究改革多部门之间相互前置的审批体制，提高审批效率，全面放宽投资领域，促进融资渠道更加多样、投融资更加便利。

深化土地管理制度改革。落实北部湾经济区土地管理制度改革先行先试的要求，深化城乡建设用地增减挂钩、集体经营性建设用地流转、城市低效土地再开发利用、缓坡荒滩等未利用地综合开发利用、土地“只征不转”等改革。深入推进征地制度改革，完善对被征地农民的合理、规范、多元保障机制，并与规范农村集体经营性建设用地流转相协调。依法实行农村集体经营性建设用地和国有土地同等入市、同权同价，统筹城镇建设用地与农村集体建设用地，统筹增量与存量建设用地，使土地利用更加集约高效、土地收益分配更加公平。

深化社会事业改革。深化教育事业改革，统筹城乡义务教育资源均衡配置，建立公办学校县（区）域内校长教师交流轮岗制度，试行学区制和九年一贯制对口招生，实施教育发展协同创新项目。鼓励和支持社会力量举办各类教育，不断扩大教育资源总量。加快医疗卫生体制改革，完善突发公共卫生事件应急和重大疾病防控机制，扎实开展新农合大病医疗保险和支付改革试点、县级公立医院改革等工作。完成食品药品监管体制改革。加快推进科技、文化、体育、就业创业、收入分配、社会保障、计划生育等社会事业改革，使社会服务更加多样化，更好地满足人民需求。

深化其他各项改革。积极参与北部湾经济区综合配套改革，争取更多的改革举措在北海先行先试，努力打造改革的先行区和试验区。加快国有企业改革，提高国有资产使用效益，积极发展混合所有制经济。推进工程建设领域的管理创新，深化工业项目配套用地指标控制等环节的改革。加强公务员队伍建设，积极推行市直行政机关科长交流轮岗制度。抓好社会治理、外向型经济发展、生态文明建设等领域的体制机制改革创新。

着力扩大开放合作。抓住国家共建21世纪海上丝绸之路、打造中国-东盟自贸区升级版，以及北部湾经济区和西江经济带“双核”驱动战略全面实施的机遇，认真落实李克强总理关于把广西建设成为西南、中南地区开放发展新的战略支点和北海要“北靠腹地、向南开拓”的重要指示精神，不断提升开放合作水平。主动融入泛北部湾区域经济合作，努力实现对东盟国家开放合作的新突破。积极争取在北海建立国家级海洋研究所，建设海洋经济合作试验区。加快推进与东部发达地区、港澳台地区的交流合作，积极承接产业转移。加强与西南、中南腹地的交流与合作，进一步拓展辐射服务空间。推进龙港新城（香港产业园）建设，打造开放合作新平台。加快国家科技兴贸创新基地(电子信息)、国家级加工贸易梯度转移重点承接地、国家外贸转型升级专业型示范基地(水产品)、自治区外贸转型升级示范基地(皮革制品) 建设。调整优化出口结构，加快发展对外贸易，外贸进出口总额达到30亿美元、增长15%。完善口岸基础设施，优化口岸通关服务，争取国家尽快批准铁山港口岸正式对外开放。

(二)加快新型工业化跨越发展，强化城市持续繁荣的产业支撑。

认真落实推动新型工业化跨越发展的决策部署，坚持走新型工业化道路，以加快打造千亿元产业为重点，大力发展先进制造业，积极培育战略性新兴产业，优化提升传统产业。完成工业投资332亿元，增长10%。

加快形成工业发展新格局。以北海组团和铁山港(龙潭)组团为两核，以北海工业园区、出口加工区、高新技术产业园区、铁山港(临海)工业区、合浦工业园、龙港新城(香港产业园)、北海国家（海洋）农业科技园区暨北海海洋产业科技园区为重点，以325国道北海段沿线地带为补充，加快构建“两核七园一带”工业发展新格局，推动企业向园区集中，促进工业集约发展。

加快打造三大千亿元产业。坚持扩大总量、提高质量、优化结构、完善配套，促进电子信息、石油化工、临港新材料三大产业集群化和规模化发展，力争分别完成产值750亿元、350亿元和230亿元。开工建设北海工业园区电子信息配套产业园，建成惠科电子科技产业园一期、朗科国际存储产业园二期等项目，支持建兴光电、冠捷科技、国钰电子等企业扩能扩产。规划建设软件产业园，大力发展软件研发、文化创意、动漫设计等服务业，形成软件与信息服务业集群。紧紧围绕打造铁山港石化产业基地，加快推进北海炼化产品质量升级改造、广西(北海)LNG项目和石化成品油码头建设，完成铁山港铁路支线、中石化铁路专用线和固体废物填埋场建设并投入使用，建成投产和源化工、中航化工、新鑫碳四等一批配套产业项目，继续做好千万吨级炼化一体化项目前期工作。加快推进诚德新材料扩能改造和冷轧工程建设，引进一批配套企业，发展形成下游产业，打造不锈钢加工和新型材料产业集群。

加快发展海洋经济和其他优势产业。大力开发海洋资源，扎实打造海洋强市。推进北海国家（海洋）农业科技园区暨海洋产业科技园区建设，加快引进国内领军海洋科研机构和高新技术企业，形成高端海洋产业集群。加快发展以水产品为重点的农产品加工业，培育形成一批品牌，提高产品附加值。巩固发展林纸、能源和船舶修造等产业，重点推进林纸一体化、神华国华广投北海能源基地、和润粮油加工、国投北海电厂二期等一批项目建设。全面落实国家消化过剩产能的要求，加快淘汰落后产能，推进消化过剩产能。

提升配套服务水平。加大财政对工业发展的支持力度，从今年起市财政每年安排1亿元以上的专项扶持资金，并结合财力增长情况逐年增加。力争新增规模以上企业15家、亿元企业8家以上。推进铁山港(临海)工业区交通、电力、环保基础设施和生活配套建设，完成出口加工区B区一期工程建设、实现封关运行，继续推进北海工业园区和高新技术产业园区升级，提升信息、物流、金融、政务及生活配套等服务水平。促进工业化和信息化深度融合，推进企业信息化改造。加快以企业为主体的技术创新步伐，新增自治区级企业技术中心和研发中心各3家以上。

(三)加快推进新型城镇化和农业现代化，扎实构建城乡一体化发展格局。

按照建设生态宜居文明城市和统筹城乡发展的要求，加强城乡基础设施建设，发展特色现代农业，完善城镇管理服务，加强生态文明建设，扎实推进以人为本的新型城镇化，为在广西率先实现城乡一体化发展奠定更加坚实的基础。城镇化率提高到54%，农林牧渔业总产值增长5%以上。

加强城乡规划引导。按照促进生产空间集约高效、生活空间宜居适度、生态空间山清水秀的总体要求，形成生产、生活、生态空间的合理结构，增强城市综合承载力。抓紧完成城市总体规划修编，加强控制性详细规划动态调整，推进重大专项规划编制，加快完善以总规为核心、控规为主体、专项规划为保障、重点区域设计为支撑的城乡规划体系。抓好乡镇规划和建设管理机构建设，加强村镇规划编制指导，推进村镇规划集中行动，抓好历史文化名镇名村保护。加强用地审核把关，提高城镇建设用地和产业项目用地集约化程度。加强规划执法，严厉打击城乡违法用地用海、圈占岸线滩涂和违法建设行为。

加快农业基础设施建设。推进农田水利建设三年攻坚计划，抓好海河堤标准化、农田水利、病险水库除险加固、中小河流治理建设，新增水土流失综合治理面积20平方公里，重要水功能区主要水质达标率达100%。统筹推进城乡自来水联网建设，实施自来水村村通达工程，从今年起用3年的时间全部解决农村饮水安全问题，在广西率先实现自来水通达所有行政村的目标。加强农村公路建设和养护管理，建设农村公路160公里。加强农村污水排放、垃圾清运设施和渔港改造建设，改善农民、渔民生产生活条件。开展贫困村整村推进扶贫攻坚，提高扶贫工作的精准性、有效性、持续性。加快农产品物流园建设，继续实施放心粮油工程，提升农产品流通水平。推进农业生产机械化，抓好水稻、甘蔗、花生全程机械化示范基地建设。

大力发展特色现代农业。稳定粮食生产，确保播种面积达到121万亩以上，提高粮食安全水平。加快发展高效设施农业，力争面积达到15万亩。加快“米袋子”、“菜篮子”工程建设，确保秋冬种面积70万亩以上。积极推广农业新技术、新品种，提高农业科技水平。扶持一批农业产业化龙头企业，新增农民专业合作社35家，创建一批特色鲜明的“一村一品”专业村。积极发展远洋渔业，加快发展珍珠和名优水产品养殖。扶持建设一批无公害标准化生产基地，加强农产品质量和安全监管。大力发展名贵花卉种植，推进罗汉松产业化发展，培育壮大特色庭院经济、林下经济。加快发展外向型农业，提高农业开放合作水平。加大强农惠农富农政策力度，扶持发展乡镇中小企业、休闲农业、乡村旅游，加强对失地、失海农民的就业技能培训，引导和支持农民、渔民转产转业，多渠道增加收入。

全面提升城镇建设治理水平。结合产业园区的发展，加快推进产城融合，建设功能齐全、配套完善、产业集聚的新城区。加快廉州湾区域和铁山港东岸开发建设，推进合浦县城与主城区一体化发展，稳步有序扩大城市规模。继续打通市区一批道路，完善“七纵七横”主干道。推进金海岸大道、重庆路、西藏北路、竹林大道等市政道路和新一批示范街、示范路、示范小区、小街小巷改造建设，开展北部湾广场和北海大道、四川北路等重要干道的风貌整治。加强市区内涝整治，完成给排水规划编制，加快实施市区内涝整治二期工程，重点抓好迎宾大道、南珠大道、北海大道、西南大道、广东路等易涝路段整治，完成铁路北侧明渠东段、三江排洪渠整治项目，力争用3年时间基本完成市区排涝设施建设。高标准完成园博园建设，打造城市绿心，带动冯家江区域发展。落实国家宏观调控政策，促进房地产业平稳有序发展。加强保障性住房建设，推进城市棚户区、城乡风貌、农村危房和城中村改造，抓好老城区保护和改造建设，加快侨港片区改造，改善城乡居民居住条件。合理规划建设一批公厕、停车场、小绿地、便民市场、健身场所，改造提升一批公园，提高生活宜居度。坚持优先发展城市公共交通，优化路线和站点设置，推动城乡公交一体化发展。加强出租车运营监管，进一步提升车辆档次、规范运营秩序、提高服务水平。严厉打击“四车”非法投入营运、上路行驶、占道经营、生产销售行为，净化市区交通环境。加强交通疏导管理，综合治理交通违法现象，提升道路安全畅通水平。加强和规范城市综合管理，坚持疏堵结合，推行人性化管理，提升文明执法水平。实施大县城战略，有重点地发展小城镇，培育经济强镇、特色名镇，加快构建科学的城镇体系。抓好新型城镇化试点建设，完善城镇基础设施和公共服务设施，增强辐射服务功能，促进人口和产业集聚，加快农业转移人口市民化。科学推进村庄建设改造，注重保留原始风貌，加快社会主义新农村建设。

深入推进生态文明建设。加快国家环保模范城和国家海洋生态文明示范区创建工作，深入开展“美丽广西·清洁乡村”活动，推进美丽北海建设。完成红坎污水处理厂二级处理二期工程、大冠沙污水处理厂和西南大道污水收集系统建设，逐步完善市区污水管网，推进沿海岸线污水截流。编制生活垃圾处理设施建设规划，推行生活垃圾分类减量处理，开工建设污泥集中处理等项目。积极参与“绿满八桂”、“北部湾绿色屏障”建设，实施生态修复和海岛、海岸带综合整治工程，加大农村沼气池建设力度，促进生态涵养保护。加强银滩、涠洲岛等重要区域和水资源、滨海湿地、红树林等重要资源保护，严厉打击盗采海河砂、珊瑚、高岭土和电捕鱼等非法行为。开展牛尾岭水库和湖海运河综合整治，提高城市供水安全水平。推行畜禽养殖分区管理，划定主城区大畜禁养区。深入推进环境安全隐患清查整治，切实防范环境安全事件发生。逐步推行机动车环保标志管理，全面完成节能减排任务。加强环保能力建设，加大环保执法力度，提高环境监测和预警应急水平，尽最大努力守护北海的碧海蓝天，留住“中国最大天然城市氧吧”的美誉。

(四)加快旅游产业跨越发展，全面繁荣发展第三产业。

认真落实推动旅游产业跨越发展的决策部署，扎实打造北部湾国际旅游度假区核心城市、广西主要旅游集散地和国内一流、国际知名的滨海旅游休闲胜地，带动第三产业全面发展。接待国内游客1700万人次、增长13%，完成社会消费品零售总额192亿元、增长15%。

优化旅游发展格局。发挥比较优势，以北海银滩和涠洲岛为双核，以北海老城和合浦系列景区为两翼，加快构建“双核两翼”的旅游协调发展格局。积极引进大企业大财团整体开发涠洲岛，加快供水、供电、环岛路、港口码头等基础设施，以及火山国家地质公园鳄鱼山门景区、珊瑚博物馆等景区景点项目建设。启动冠山海o欢乐海岸（二期）、珊瑚海海洋乐园建设，争取启动国际游艇俱乐部建设，逐步将银滩西区打造成为功能完善的高端滨海度假休闲娱乐区。推进银滩和鳄鱼山景区创5A，力争完成北海老城、金海湾红树林景区创4A，编制竹林旅游综合休闲区总体规划，推进星岛湖、山口红树林景区保护开发，全面提升重要景区景点的等级档次。

推动旅游产业转型升级。积极培育发展滨海休闲等旅游业态，着力打造海岛旅游、滨海度假、海上邮轮、避寒养生、海洋运动、海丝风情等六大旅游品牌。加快高星级度假酒店建设，完成富丽华大酒店建设，开工建设冠岭山庄（二期）、涠洲西角旅游综合体等一批高星级酒店，推动形成银滩和涠洲岛亲海度假酒店群。扶持发展中越海上旅游航线，积极做好延伸拓展工作。编制全市游艇码头规划，建设滨海特色城市港湾，大力发展邮轮游艇经济，积极引进水上飞机。促进旅游与文化融合发展，推进合浦汉文化公园、曲樟客家生态文化旅游区、白龙珍珠城建设，开辟近代西洋建筑文化旅游线路，推动《碧海丝路》等优秀剧目走向市场、增创效益。

深入开展旅游环境综合整治。完善旅游、公安、交通、文化、物价、工商、食品药品监管等部门联合执法机制，加大执法和监管力度，规范市场秩序，提升服务质量，维护游客合法权益，进一步提高游客满意度。加强旅游整体宣传营销，充分利用手机短信、微博微信、微电影、旅游网站等平台和形式进行旅游推介，提升北海旅游形象。

大力发展现代物流业。全面推进港口、铁路、航空、公路基础设施建设，加快构建互联互通的国际大通道。开工建设北海公共客运码头、铁山港5号及6号泊位、铁山港航道二期扩建及三期项目，推进铁山港7-17号泊位前期工作，继续培育扶持海运定期班轮航线，加快打造亿吨大港。开工建设铁山港铁路物流中心和1-4号泊位铁路专用线，推进铁山港(临海)工业区内其他铁路项目，争取开工建设合浦至湛江铁路，打造西南出海铁路大通道。继续培育航空市场，力争完成机场旅客吞吐量100万人次。开工建设贵港至合浦高速公路，协调推动广西滨海公路北海段早日开工建设，推进北铁一级公路改造，完善公路主干路网。抓住南北高铁等一批重大交通项目投入运营的有利时机，加快培育发展物流产业，规划建设大型专业批发市场，引进大型物流企业，建设现代物流园区，服务产业发展和城市建设。

加快繁荣发展其他服务业。大力发展电子商务、连锁经营和物流配送等现代流通业态，积极申报国家级电子商务示范基地。加快东盟国际商贸城、红星美凯龙城市综合体等商贸项目，以及北海老城、银滩四号路等商业步行街建设。大力发展会展经济，策划建设北部湾国际贸易展览中心，积极通过市场化运作举办特色传统节庆展会活动，办好第四届广西园博会、北海国际珍珠展和银滩旅游文化节。推动科技、家政、养老、中介等服务业加快发展。

(五)全面加强社会建设，努力提高人民生活质量。

加快推进社会事业改革发展，繁荣发展先进文化，切实保障和改善民生，加强和创新社会管理，确保社会和谐稳定。

统筹发展各项社会事业。坚持创新驱动发展战略，提升自主创新能力。提高全民科技素质，抓紧筹建市科技馆。坚持优先发展教育，继续办好学前教育，加强义务教育中小学校标准化建设，进一步解决义务教育“大班额”问题，尽力让每个孩子都能上到“好学堂”，九年义务教育巩固率提高到96%，学前三年毛入学率提高到77.5%，高中阶段教育毛入学率提高到91%。新建或改扩建一批中小学校、幼儿园，年内新增86个班、4000个学位。积极探索普通高中特色化多样化发展，大力发展职业教育和特殊教育，推进大学园区周边公共设施配套建设。完成第二人民医院迁建主体工程，加快推进广西海难紧急医学救援中心、市人民医院异地扩建、儿童医院新建前期工作，继续实施120急救网络体系建设，做好迎接自治区卫生城市复审工作。继续大力引进优质教育、医疗等社会事业资源，提高城市竞争力。加强食品药品安全监管，在每个乡镇、街道设立食品药品监督管理所，保障好群众餐桌上的安全。加快体育基础设施建设，广泛开展全民健身活动。推进人口计生基本公共服务全覆盖，提高出生人口素质。保障妇女儿童合法权益，鼓励发展老龄服务和慈善事业，推进老龄服务体系建设，提高农村养老服务水平。继续支持海防、国防动员和后备力量建设等工作。推动档案、地方志、人防、消防等其他各项社会事业加快发展。

推动文化大发展大繁荣。深入发挥北海作为国家历史文化名城的优势，完善文化管理体制，建立健全现代文化市场体系和公共文化服务体系。开工建设市群艺馆新馆，建成园博园“天天演”基地等一批文化项目，做好市城市规划馆、海上丝绸之路博物馆、综合档案馆、北部湾国际文化广场等文化项目前期工作，争取尽快开工建设。加强市场监管，强化“扫黄打非”执法，促进文化市场繁荣发展。深入挖掘、保护和开发历史文化资源，加强重点文物保护单位维修，推进“海上丝绸之路”申遗、合浦汉墓群申报国家考古大遗址公园工作。繁荣文艺创作，打造文化精品，加强对外文化交流。

加快健全社会保障体系。把促进就业工作摆在更加突出的位置，鼓励多渠道多形式就业，提升高校毕业生就业质量，全面实施创业培训进社区、进乡镇、进校园“三进计划”。健全覆盖城乡居民的社会保障体系，整合城乡居民基本养老保险和基本医疗保险制度，加强养老保险基金征收，健全社会保障管理体制和经办服务体系。加强和改进低保工作，努力使困难群众生存有尊严、生计有保障、生活有盼头。健全社会救助和残疾人社保服务体系，保障农民工合法权益。做好价格调控工作，保持物价总水平稳定。继续实施城乡居民收入倍增计划，努力提高人民生活水平。

努力创新社会治理。改进社会治理方式，提高社会治理水平。强化政府社会管理服务职能，加强对社会组织和网络舆论的引导，拓宽群众利益诉求的表达渠道，维护群众合法权益，促进社会公平正义。加强社会治安综合治理，依法严厉打击“两抢一盗”、“黄赌毒”、传销等违法犯罪活动。强化城乡社区自治功能，加快乡镇（街道）社会管理服务中心和社区综合管理服务平台建设。全面落实政府安全生产监管责任和企业主体责任，抓好安全隐患排查和整改，加大对水陆交通、烟花爆竹等重点行业和领域的“打非治违”力度。深入开展矛盾纠纷排查调处，加强信访案件源头治理。不断推进应急管理规范化建设，有效预防和应对各类突发事件。

继续实施为民办实事工程。筹措资金24.5亿元，实施社保、健康、教育、强基、安居、农补、生态、文体、城建、平安惠民等10项为民办实事工程。

三、全面建设人民满意的政府

各位代表！面对科学发展新要求和全市人民新期待，我们将以改革创新的精神加快政府自身建设，不断提升真抓实干、把事干成、造福百姓的能力和水平，进一步树立为民、务实、清廉的政府形象。

一是推进科学施政，进一步转变政府职能。正确处理市场与政府的关系，努力用好市场这只“看不见的手”，充分发挥市场在资源配置中的决定性作用；切实用好政府这只“看得见的手”，更好地为市场主体和产业结构调整服务，推动政府职能向经济调节、市场监管、公共服务、社会管理、环境保护等方面转变，做到不缺位、不越位、不错位。树立正确的发展观和政绩观，准确考核科学发展、全面发展的成效。深化行政体制改革，创新行政管理模式，推进扩权强县、扩权强镇，提高县区、乡镇发展能力。推进事业单位改革，扩大政府购买公共服务范围。继续深化行政审批制度改革，全面推行行政审批“三集中”，逐步实现“审管分离”，重在加强事中、事后的服务和监管。最大限度减少审批事项，该取消的坚决取消，该下放的彻底下放，为企业“松绑开路”。进一步优化审批流程，压缩审批时限，提高审批效率。依托电子政务平台，加快构建覆盖市县镇村的四级政务服务网络体系，努力让群众少跑路就能办好事，不求人就能办成事。

二是推进依法行政，进一步规范政府行为。牢固树立依法治市理念，用法律思维建设现代法治政府，坚持在法治环境下履行好政府职能。深化行政执法体制改革，进一步解决权责交叉、多头执法问题。强化食品药品、安全生产、环境保护、劳动保障等重点领域基层执法，加强对县区及乡镇行政处罚裁量权的规范化建设。加强行政执法监督，严格规范公正文明执法。自觉接受市人大及其常委会的依法监督和市政协的民主监督，高质量做好人大代表建议和政协提案办理工作。认真听取各民主党派、工商联、人民团体和无党派人士的意见建议，主动接受公众监督和舆论监督。深化政务公开，逐步建立权力清单制度，做到决策公开、管理公开、服务公开、结果公开。

三是推进务实勤政，进一步改进工作作风。深入贯彻落实中央“八项规定”精神，认真开展党的群众路线教育实践活动，全面加强服务政府建设。突出抓好政府机关作风整改，针对政府部门及其工作人员在履行职责、服务企业和群众方面存在的突出问题，剖析曝光典型，加强教育培训，强化管理监督，健全各项制度，形成长效机制，争取用1年的时间取得明显成效。今明两年集中开展“企业发展服务年”活动，建立市政府领导与重点企业“直通车”沟通机制，完善领导联系重大项目制度，加大协调服务力度，优化企业发展环境，促进企业发展壮大。深入落实领导干部带头深入基层调查研究、联系服务群众机制，坚持沉下心、俯下身，深入基层跟老百姓交朋友，和群众“零距离”。大兴务实之风，力戒形式主义，多办雪中送炭的实事，少做锦上添花的虚功，坚决杜绝劳民伤财的“形象工程”和“政绩工程”。深化会议公文制度改革，继续精简会议和文件，严格控制以政府名义举办全市性会议和大型活动。加强绩效管理，强化督查督办，加大行政问责力度，严肃查处行政不作为、慢作为、乱作为，不断增强政府公信力和执行力。

四是推进廉洁从政，进一步提升政府清廉形象。按照“干部清正、政府清廉、政治清明”的要求，严格落实党风廉政建设责任制，推进惩治和预防腐败体系建设，把廉政建设落实到政府工作的每一个环节。加强行政监察和审计监督，强化领导干部经济责任审计。全面推进公共资源交易监管体制改革，构建统一规范的交易平台，使各类公共资源交易活动统一进场交易、进行管理、接受监督。严格落实党政机关厉行节约反对浪费条例、党政机关国内公务接待管理规定等政策规定，把有限的资源和财力用在推动发展急需的地方、用在改善民生最迫切的事情上。我们一定时刻做到自重、自省、自警、自励，担当清正的公务员，建设清廉的政府，对人民负责，让人民放心！

各位代表! “空谈误国，实干兴邦”，唯有脚踏实地、扎实苦干才能将宏伟蓝图变为美好现实。让我们紧密团结在以习近平同志为总书记的党中央周围，在自治区党委、政府和市委的正确领导下，解放思想，改革创新，开拓奋进，扎实工作，为加快在广西率先全面建成小康社会步伐而努力奋斗！
